# Intersection of Inflammation and Herbal Medicine in the Treatment of Osteoarthritis

**DOI:** 10.1007/s11926-012-0288-9

**Published:** 2012-09-18

**Authors:** Ali Mobasheri

**Affiliations:** Musculoskeletal Research Group, School of Veterinary Medicine and Science, Faculty of Medicine and Health Sciences, The University of Nottingham, Sutton Bonington Campus, Sutton Bonington, LE12 5RD UK

**Keywords:** Osteoarthritis, Rheumatic diseases, Musculoskeletal complaints, Inflammation, Herbal medicine, Ethnopharmacology, Ayurvedic medicine, Nutrigenomics, Clinical trials, Articular cartilage, Synovium, Mesenchymal stem cell, Phytochemicals, Flavonoids, Catechins, Treatment

## Abstract

Herbal remedies and dietary supplements have become an important area of research and clinical practice in orthopaedics and rheumatology. Understanding the risks and benefits of using herbal medicines in the treatment of arthritis, rheumatic diseases, and musculoskeletal complaints is a key priority of physicians and their patients. This review discusses the latest advances in the use of herbal medicines for treating osteoarthritis (OA) by focusing on the most significant trends and developments. This paper sets the scene by providing a brief introduction to ethnopharmacology, Ayurvedic medicine, and nutrigenomics before discussing the scientific and mechanistic rationale for targeting inflammatory signalling pathways in OA by use of herbal medicines. Special attention is drawn to the conceptual and practical difficulties associated with translating data from in-vitro experiments to in-vivo studies. Issues relating to the low bioavailability of active ingredients in herbal medicines are discussed, as also is the need for large-scale, randomized clinical trials.

## Introduction

The global incidence of age-related diseases of bone, joint, and muscle is steadily rising, seriously affecting the health of millions of people across the world. According to the United Nations (UN) [1] and the World Health Organization (WHO) [2] musculoskeletal, rheumatic, and arthritic conditions are leading causes of morbidity and disability throughout the world, and result in enormous healthcare expenditure and loss of work [3] (sources: The Arthritis Foundation (AF) and WHO [4–6]). The most common and important form of arthritis is osteoarthritis (OA), also known as osteoarthrosis or degenerative joint disease (DJD). OA is the most common type of degenerative joint disease. It is the major cause of pain and disability affecting the elderly [7]. A 2005 study in the USA estimated that OA is one of the top five causes of disability amongst non-hospitalized adults (source: Centers for Disease Control and Prevention, USA (CDC) [8]). According to estimates from the National Institute of Arthritis and Musculoskeletal and Skin Diseases (NIAMS) more than 20 million Americans currently suffer from OA [9]. In 2006, it was estimated that around 35 to 40 million Europeans had OA. It is expected that by 2030, 20 % of adults will have developed OA in Western Europe and North America. Therefore, OA is expected to be a heavy economic burden on healthcare systems and community services in Europe and the rest of the world.

Advancing age is a major risk factor for development of OA. There is radiographic evidence of OA in at least one joint in most of the human population aged 65 or over. Although OA is rare in people under 40, it becomes much more common with age. The end stage treatment for OA is surgery, either to modify or replace the joint. With increasing life expectancy, growth of the elderly population, and an alarming escalation of chronic, inflammatory, and age-related conditions (for example OA), there is increased demand for new treatments and preventative approaches.

Although OA is primarily associated with aging, there are other important contributing factors [10]. These include genetics, underlying anatomical and orthopaedic disorders (i.e. congenital hip dislocation), obesity, underlying inherited or acquired metabolic disease, endocrine disease, various disorders of bone turnover and blood clotting, joint infection, crystal deposition, previous rheumatoid arthritis (RA) or a history of joint trauma, repetitive use, muscle weakness, or joint instability. The mechanical and metabolic alterations that occur in obesity, with the pro-inflammatory factors produced by white adipose tissue in the chronically overweight, are thought to be major factors in the progression of the disease [11].

Symptoms of OA in the most frequently affected joints include pain, stiffness and limited mobility, swelling, and, occasionally, warmth. These manifestations are highly variable, depending on joint location and disease severity. OA can affect any synovial joint but it commonly affects large load-bearing joints such as the hip and knee. The disease is often thought of as being a result of daily wear and tear of the joint and, indeed, the accumulation of microtrauma to cartilage and bone contribute to pathogenesis. The most prominent anatomical feature is the progressive destruction of articular cartilage [12]. However, OA is a disease involving not only articular cartilage but also the synovial membrane, subchondral bone, and peri-articular soft tissues [13]. Inflammation of the synovium occurs in both the early and late phases of OA and is associated with alterations in the adjacent cartilage. This inflammatory synovitis is qualitatively highly similar to that seen in RA. Catabolic and proinflammatory mediators, for example cytokines, nitric oxide (NO), prostaglandin E2 (PGE_2_), and neuropeptides are produced by the inflamed synovium and alter the balance of cartilage matrix degradation and repair. These events lead to excess production of the proteolytic enzymes responsible for cartilage breakdown [14]. Cartilage alterations induce further synovial inflammation, creating a vicious circle. The progressing synovitis will then exacerbate clinical symptoms and joint degradation in OA [14].

**Fig. 1:**
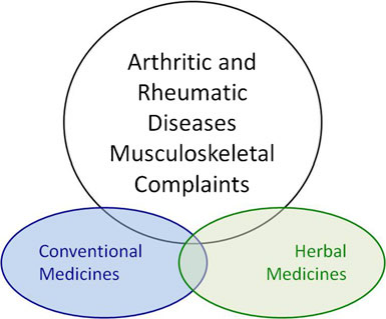
Venn diagram indicating the overlap between conventional and herbal medicines in the treatment of arthritic, rheumatic, and musculoskeletal diseases. Some of the drugs in our pharmacopoeia (or their derivatives) have a long established history in ethnopharmacology and have been used for centuries

This article is a narrative review of herbal medicines for OA and the challenges and opportunities facing this area of research. Although most of the focus is on OA, the material discussed is also relevant to other types of rheumatic disease and musculoskeletal complaints for which pathogenic mechanisms of different conditions overlap. This paper summarizes the personal views and perspectives of a basic scientist working in the area of cartilage biology and inflammatory signalling in chondrocytes. For thousands of years human beings have used and refined herbal medicines for treatment a variety of inflammatory diseases. Many ancient civilizations and cultures used herbal extracts for their medicinal effects. Indeed, many of the anti-inflammatory drugs in our current pharmacopoeia have long established roots in ethnopharmacology (Fig. 1). This paper will review the most up-to-date information and current trends and topics in this area. The author has tried to make the review as mechanistic as possible, highlighting the scientific rationale for targeting inflammatory pathways in osteoarthritis (OA) by use of herbal medicines. This article will also attempt to draw attention to the conceptual and practical difficulties associated with translating data from in-vitro models. Many naturally occurring anti-inflammatory compounds in plants are poorly absorbed in the gastrointestinal tract or rapidly metabolized and excreted by the liver and the kidneys resulting in low bioavailability. Consequently, in-vivo data and large-scale, randomized clinical trials supporting the theoretical reasons for using herbal medicines are largely lacking.

## Why Do We Use Herbal and Complementary Medicines?

There is currently no effective pharmacotherapy capable of restoring the original structure and function of the damaged cartilage and other synovial tissues in OA or, indeed, any other form of arthritis. Apart from analgesics, limitations to conventional medical management of OA indicate a genuine need for novel, safe and effective treatments for OA patients. Herbal medicines have the potential to provide a solution to this problem.

The growing interest in herbal medicines and nutraceuticals may reflect a general and increasing disenchantment with traditional medicine. This could be because of several factors: conventional treatment may not be working as well as patients would like; patients want greater relief of symptoms and/or disability; they have issues with side-effects of pharmaceutical treatment; they wish to reduce some of the stress that comes from living with a chronic illness and want to cope better; they believe that herbal and complementary therapy is safer and more “natural”; and they are influenced by the widespread advertising and attractive claims that are made for many natural products. Another reason why people use herbal and complementary medicines could be the corruption and gradual degradation of the classic patient–physician relationship by “managed care” and other cost cutting trends in the practice of medicine. With chronic and incurable diseases, for example OA, there can be considerable frustration at the inability of modern scientific medicine to treat the disease. Consequently, there is an increase in the patient-driven search for alternative treatments.

## Ethnopharmacology

Ethnopharmacology is the systematic study of the use of herbal and medicinal plants by specific cultural groups. As a discipline, ethnopharmacology is new and rapidly developing. Today, it is used to designate a field of specialization that focuses on medicinal, psychoactive and toxic plants, fungi, or even animals that are used by people all over the world, and for research on nutraceuticals and food supplements. The ethnopharmacological approach is unique in pharmacology and the natural sciences in that it also requires input from the social and cultural sciences. In summary, ethnopharmacology is a multidisciplinary and expanding field of study with its own scientific society (The International Society for Ethnopharmacology [15]) and peer-reviewed scientific journals dedicated to the discipline [16, 17].

## Ayurvedic Medicine

Ayurvedic medicine (also called Ayurveda) is one of the world’s oldest medical systems. It originated on the Indian subcontinent and has evolved there for thousands of years. In the United States, Ayurvedic medicine is considered to be a Complementary and Alternative Medicine (CAM). It includes the use of herbal medicines, massage, and specialized diets alone or in combination. Ayurvedic medicine may involve the use of products such as spices, herbs, vitamins, proteins, minerals, and metals (e.g., iron, zinc), often in mixtures prepared for the individual patient. The products are commonly sold on the Internet or in stores and are represented as “Indian” or “South Asian”. However, consumers should be aware that Ayurvedic products are not reviewed or approved by the US Food and Drug Administration (FDA [18]) before marketing. Most Ayurvedic products are marketed either for drug uses not approved by FDA or as “dietary supplements”. Consumers should be especially cautious when purchasing these products on the Internet, because the presence of metals such as lead, mercury, or arsenic in some Ayurvedic products makes them potentially very harmful [19].

## “Nutrigenomic” Treatments for OA

Nutrigenomics is the study of the effects of foods and food constituents on gene expression [20]. This field of study has emerged because of the realization that the health effects of food-derived substances start at the molecular level [20, 21]. Therefore, nutrigenomics is a form of personalized nutrition that involves designing diets to fit an individual’s genetic makeup, considering genetic variation, allergies, and intolerances [22]. Changes in gene expression result in changes in the proteome and metabolome and, consequently, result in an altered metabolic state, which may have beneficial health effects. An important objective of nutrigenomic research is defining the relationship between genes and nutrients from basic biology to clinical states. We often overlook the fact that nutrigenomics and systems biology apply the same set of tools and technology. Systems biology approaches are applied in nutritional research to describe the physiological responses of culture models, experimental animals, and human subjects by exploiting datasets which focus on biochemical pathways, molecular targets for therapy, and potential biomarkers. The nutrigenomics approach extracts relevant differences, which become leads for further hypothesis-driven and mechanistic research. One of the objectives of research into herbal and alternative medicines is to optimize health and prevent or delay disease [23••]. Research that targets specific aspects of the main drivers of health (metabolism, oxidation, inflammation, and stress responses) may be instrumental in creating knowledge for maintaining health and preventing disease through nutrition [23••].

## Proinflammatory Cytokines in OA

Osteoarthritis (OA) is characterized by degeneration of articular cartilage, limited intra-articular inflammation with synovitis, and changes in peri-articular and subchondral bone [13]. Proinflammatory cytokines are major mediators of inflammatory responses. These proteins are intimately involved in the pathogenesis of OA [13]. They cause synovial, cartilage and bone changes during disease progression. Proinflammatory cytokines are mainly produced in the synovium, predominantly by the synovial macrophages that drive the inflammatory and destructive responses in OA [24]. These cytokines are thought to diffuse through the synovial fluid into the cartilage where they stimulate chondrocytes and synoviocytes to increase cytokine production and the production of degradative proteases. The intimal cells of the synovium are most significant in the production of cytokines that cause inflammation [25]. The main proinflammatory cytokines involved in the pathogenesis of OA are TNF-α and IL-1β, which act on synoviocytes and chondrocytes via specific interactions with cytokine receptors on the cell surface. These receptors include the IL-1β receptor, the IL-1 receptor type I, the TNF-α receptor, and the TNF-R55 receptor, which are highly expressed in synovial fibroblasts [13, 26].

## Proinflammatory Signalling and Activation of NF-κB

NF-κB (nuclear factor-κB) is a rapidly acting primary transcription factor involved in cellular responses to inflammation and stress. In OA, NF-κB can be initiated by a host of stress-related stimuli, including proinflammatory cytokines, excessive mechanical stress, and extracellular matrix degradation products [27••]. In unstimulated cells, NF-κB dimers are sequestered inactively in the cytoplasm by a protein complex called inhibitor of κB (IκB). IκB inactivates NF-κB by masking the nuclear localization signals (NLS). Activation of NF-κB occurs via degradation of IκB, a process that is initiated by its phosphorylation by IκB kinase (IKK). Phosphorylated IκB becomes dissociated from NF-κB, unmasking the NLS. Phosphorylation also results in IκB ubiquitination and targeting to the proteasome. Once phosphorylation of IκB and unmasking of the NLS has occurred, NF-κB enters the nucleus to regulate gene expression. NF-κB turns on expression of IκB, forming a negative feedback loop. Targeted strategies to prevent unwanted or excessive NF-κB activation are the focus of current OA research. Work in this area is focused on the use of highly specific drug modalities, small interfering RNAs (siRNAs), or other biological inhibitors [27••]. Some of these biological inhibitors may come from natural products, plants, or herbs. It has been suggested that targeting NF-κB in OA will need to consider the undesirable systemic effects of synthetic drugs [27••].

## Naturally Occurring NF-κB Inhibitors

Currently available drugs for OA are associated with unwanted side effects and are expensive. Naturally occurring compounds capable of blocking NF-κB may be promising therapeutic agents for treatment of OA. In recent years, there has been a significant increase in the application of nutrigenomics in biomedical research. This has resulted in substantial growth in phytopharmacology and renewed interest in naturally occurring plant-derived compounds as potential therapy for a variety of immune-related conditions. Several herbal medicines have been investigated for their anti-inflammatory and indirect MMP inhibitory capabilities. These include phytochemicals and flavonoids and catechins from green tea, rosehip, curcumin, and resveratrol (reviewed in Ref. [28]).

## Rosehip (*Rosa canina*)

Rosehip powder is extracted from the seeds and husks of the fruits of a sub-type of *Rosa canina* and has been used extensively in traditional medicine in tea, taken 3 or 4 times per day. Rosehip powder also contains substantial amounts of vitamin C. In-vitro, rosehip preparations have anti-inflammatory and anti-oxidative properties, and have been shown to inhibit expression of iNOS, IL-1α and MMP-9, and IL-1β-induced ADAMTS-4, MMP-1, MMP-13, IL-1α, and IL-8 in chondrocytes. The likely mechanism of action is via the specific galactolipid constituent. One proprietary preparation of this glycoside of mono and diglycerol is now a patented compound that claims to temporarily relieve arthritic pain. A meta-analysis of randomized controlled trials (RCTs) of a *Rosa canina* (rosehip) powder preparation for symptomatic treatment of OA was carried out to estimate its empirical efficacy as a pain-reducing compound [29]. Although the effect size was small, the meta-analysis revealed that rosehip powder does reduce pain and results in a statistically significant reduction in the use of analgesics. The study concluded that although the efficacy and safety of rosehip needs evaluation and independent verification, a large-scale/long-term clinical trial is justified [29]. This study led to a recently registered clinical trial (ClinicalTrials.gov Identifier: NCT01430481 [30]) entitled: “Rosehip Powder for Knee Osteoarthritis” at the Frederiksberg University Hospital in Denmark to compare two different rosehip products, one of which is tested in two different doses, in a non-inferiority design. The clinical trial is an interventional (parallel assignment), randomized, double blind safety and efficacy study comparing different preparations and dosages of rosehip powder in patients with painful knee OA.

## Curcumin

Curcumin (diferuloylmethane) is a polyphenol phytochemical found in the spice turmeric, derived from rhizomes of the plant *Curcuma longa*. Turmeric has been used in Ayurvedic medicine for heartburn (dyspepsia), stomach pain, diarrhoea, intestinal gas, stomach bloating, loss of appetite, jaundice, liver problems, and gallbladder disorders. Curcumin has been shown to have potent antioxidant, anti-inflammatory, and anti-catabolic effects. It has been used as an anti-inflammatory treatment in traditional Chinese and Ayurvedic medicine. We recently reviewed the biological action of curcumin on cartilage and articular chondrocytes [31•]. Curcumin antagonizes crucial catabolic effects of IL-1β signalling that are known to contribute to the pathogenesis of OA. Research in our laboratory has shown that curcumin protects chondrocytes from the catabolic effects of IL-1β, including upregulation of MMP-3 and suppression of matrix synthesis [32]. IL-1β suppresses type II collagen and β1-integrin synthesis in chondrocytes but these can be inhibited by curcumin [33]. Curcumin can also antagonize IL-1β-induced caspase-3 activation in chondrocytes. Closer examination of the effects of curcumin on inflammatory signalling in chondrocytes has revealed that this phytochemical can suppress IL-1β-induced NF-κB activation, leading to inhibition of expression of cyclooxygenase-2 (COX-2) and matrix metalloproteinase-9 (MMP-9) in articular chondrocytes [34]. Curcumin also suppresses glycosaminoglycan (GAG) release in IL-1β-stimulated cartilage explants [35]. Collectively, these results indicate that curcumin is a safe and promising herbal medicine for treatment of OA. However, its efficacy and bioavailability must be studied in greater detail in-vivo.

Work by Funk and colleagues [36] has demonstrated that turmeric extracts containing curcuminoids (compounds related to curcumin in structure and function) can prevent experimental RA. The authors used the streptococcal cell wall (SCW)-induced arthritis model, a well-described animal model of RA. Arthritic index, a clinical measure of joint swelling, was used as the primary endpoint for assessing the effect of extracts on joint inflammation. An essential oil-depleted turmeric fraction containing 41 % of the three major curcuminoids was found to be efficacious in preventing joint inflammation when treatment was started before, but not after, the onset of joint inflammation. Interestingly, a commercial sample containing 94 % of the three major curcuminoids was even more potent in preventing arthritis than the essential oil-depleted turmeric fraction when compared by total curcuminoid dose per body weight. The authors concluded that the three major curcuminoids present in the purer form are responsible for this antiarthritic effect, whereas the remaining compounds in crude extracts of turmeric may inhibit this protective effect. This study emphasised that significantly different results and observations may arise from studies that use crude extracts of turmeric and those that focus on chemically purer forms of curcumin.

There are some concerns about the cytotoxicity of curcumin in culture models of cartilage [37]. However, the concentrations used in these studies are significantly higher than the maximum concentration that can be achieved in serum and synovial fluid after a large oral dose [31•]. Moreover, the cytotoxicity data comes mainly from the C-28/I2 cell line, which is an immortalized chondrocyte cell line. The curcumin cytotoxicity observed in a transformed and immortalized chondrocyte cell line does not necessarily indicate that this compound will be toxic to untransformed primary cells and chondrocytes within articular cartilage in-vivo [38, 39••].

## Resveratrol

Resveratrol is a polyphenolic phytoalexin present in grapes, berries, and peanuts. It has been reported to have anti-inflammatory, immunomodulatory, and anti-oxidative properties. In-vitro, resveratrol has been shown to inhibit IL-1β-induced apoptosis in chondrocytes, by inhibition of caspase-3, and downregulation of the NF-κB pathway [40–43]. Resveratrol has also been shown to suppress NF-κB-dependent pro-inflammatory products, for example PGE_2_, leukotriene B4 (LTB_4_), COX-2, MMP-1, MMP-3, and MMP-13. These results suggest the use of resveratrol as an herbal medicine for treatment of OA. However, no randomized clinical trials have yet been conducted to test the in-vivo efficacy and safety of resveratrol.

## Synergistic Chondroprotective Effects of Curcumin and Resveratrol

Recent studies from our laboratories have focused on the synergistic anti-inflammatory effects of curcumin and resveratrol when used in combination. These studies have revealed that mixtures of these phytochemicals may be more effective than the individual compounds. Treatment with curcumin and resveratrol suppresses expression of the NF-κB-regulated gene products involved in inflammation (i.e. COX-2, MMP-3, MMP-9, and vascular endothelial growth factor (VEGF)) [41]. Combinations of curcumin and resveratrol inhibit apoptosis and prevent activation of caspase-3 [41]. Closer examination of the signalling pathway has shown that IL-1β-induced NF-κB activation can be suppressed directly by mixtures of curcumin and resveratrol, by inhibition of Iκκ and proteasome activation, inhibition of IκBα phosphorylation and degradation, and inhibition of nuclear translocation of NF-κB. On the basis of these results, we have proposed that combining these natural compounds may be a more useful strategy in developing herbal medicines than using the individual compounds alone.

Combining curcumin and resveratrol also activates MEK/Erk signalling. The mitogen-activated protein kinase (MAPK) pathway is stimulated in differentiated chondrocytes and is an important signalling cascade for maintenance of the chondrocyte phenotype. Activation of this pathway is thought to be required for the maintenance of chondrocyte differentiation and survival. These observations support the enhanced potential of combination therapy, with both anti-inflammatory and anti-apoptotic capabilities via inhibition of multiple components of the NF-κB pathway, to treat OA. Clearly, the concept of synergism in herbal medicine will continue to be relevant to phytopharmacology.

## Effects of Curcumin and Resveratrol on Mesenchymal Stem Cells

Mesenchymal stem cells (MSCs) are a heterogeneous subset of stromal cells that can be isolated from many adult tissues [44]. Adult MSCs can be isolated from bone marrow, marrow aspirates, skeletal muscle, adipose tissue, synovium and many other connective tissues [45]. Because of their culture-dish adherence, they can be expanded in culture while maintaining their multipotency [46]. Their multipotency is an important property that enables them to differentiate into cells of the mesodermal lineage, giving rise to a range of specialized connective tissue cells, including adipocytes, osteoblasts, chondrocytes, and tenocytes, and cells of other embryonic lineages [44]. MSCs are currently being clinically investigated as a new therapeutic for treating a variety of immune-mediated diseases [47]. Thus, they have potential applications in tissue engineering and regenerative medicine and may be an attractive option for bone, cartilage, tendon, and ligament regeneration.

Growth factors and a three-dimensional high-density culture environment are important for the differentiation of MSCs into chondrocytes, tenocytes, and osteoblasts [48–52]. IL-1β and TNF-α inhibit chondrogenesis by human MSCs via NF-κB-dependent pathways [53]. Therefore, strategies that facilitate cartilage repair under these conditions may include use of specific antagonists of IL-1β and TNF-α, or the targeting of NF-κB. Some of our recent work suggests that curcumin and resveratrol have the potential to promote chondrogenic and osteogenic differentiation of MSCs by targeting NF-κB. For example, treating MSC cultures with curcumin has been shown to suppress NF-κB, thus establishing a microenvironment in which the effects of pro-inflammatory cytokines are antagonized [54]. This facilitates the chondrogenesis of MSC-like progenitor cells co-cultured with primary chondrocytes [54]. The use of this strategy in-vitro may support the regeneration of articular cartilage in cell-based cartilage-repair techniques, for example autologous chondrocyte implantation (ACI), because cell-based repair of lesions in articular cartilage will be compromised in already inflamed joints. Resveratrol-mediated modulation of SirT-1 (a NAD(^+^)-dependent histone deacetylase) and RUNX2 (a transcription factor that encodes a nuclear protein with an Runt DNA-binding domain) promotes osteogenic differentiation of MSCs [55]. Our work also suggests that acetylation/deacetylation of RUNX2 is critical for osteogenic differentiation [55].

## Systematic Reviews of Herbal Medicines

The effectiveness of herbal medicines in the treatment of OA has been evaluated in a systematic review of randomized controlled trials of herbal medicines published by Long and co-workers in 2001 [56]. Twelve clinical trials and two systematic reviews fulfilled the authors’ inclusion criteria. The authors found promising evidence of effective use of some herbal preparations in the treatment of OA. In addition, evidence was found to suggest that some herbal medicines might actually reduce the consumption (and dosage) of non-steroidal anti-inflammatory drugs. Many of the herbal medicines reviewed seemed to be relatively safe. The authors concluded that some herbal medicines might be realistic alternatives for patients with OA.

Another systematic review of herbal therapies for OA was published in the same year by Little and Parsons [57], in the Cochrane Database of Systematic Reviews. The authors searched the databases for mainstream and complementary medicines and included any randomized trials of herbal intervention that they could find related to OA (in any language). Five studies on four different herbal interventions met the review criteria. The authors were not able to draw any firm conclusions from the single studies, but two studies indicated avocado and soybean unsaponifiables had beneficial effects on functional index, pain, NSAID consumption, and global evaluation. The investigators concluded that the evidence in support of use of avocado–soybean unsaponifiables for treatment of OA is convincing but that there is not sufficient evidence for the other herbal intervention for OA.

Soeken [58] examined the evidence from systematic reviews assessing what is known about the efficacy of selected CAM therapy for arthritis pain. Results specifically related to arthritis pain were retrieved from review articles on acupuncture, homeopathy, herbal remedies, and a selected number of nutritional supplements. Evidence was found to support the efficacy of herbal medicines including devil’s claw and avocado and soybean unsaponifiables. This study concluded that CAM therapy has potential but more high-quality research is needed for herbal medicines.

These systematic reviews have stimulated further research on herbal medicines for OA. This topic of OA research is now a thriving and expanding “niche” area. However, the presumption that herbal medicines are effective and safe is an issue that requires regular discussion and debate, and tight regulation by relevant regulatory bodies. According to the American College of Rheumatology, herbal remedies are not subjected to the same quality assurance testing that is required for prescription drugs. This has raised many concerns about the quality and safety of many of the herbal medicines that originate in the Far East. The actual contents of many herbal remedies do not necessarily match the ingredients on their label. The biggest concern at the moment is that herbal remedies can be quite toxic (or contain specific toxic components) and can interact adversely with prescription drugs.

## Clinical Trials of Herbal Medicines

A prescription medical food product containing flavocoxid and citrated zinc bisglycinate has been marketed for the clinical dietary management of the metabolic processes of OA. It was developed and formulated specifically for patients with OA. Although it is not a NSAID, nor a COX-2 selective inhibitor, it is proposed to function as an anti-oxidant, and as a dual inhibitor of the cyclooxygenase (COX) and lipoxygenase (LOX) enzymes of arachidonic acid metabolism. Flavocoxid consists primarily of the flavonoids baicalin and catechin. These flavonoids are found in commonly consumed foods, for example soy, peanuts, cauliflower, kale, apples, apricots, cocoa, and green tea. The manufacturers claim that the product provides flavonoid levels that are needed to meet the distinctive nutritional requirements of OA patients. They also claim that the flavonoid levels provided cannot be obtained by simply changing the diet. A recently conducted clinical trial (ClinicalTrials.gov Identifier: NCT00928837 [59]) entitled: “Study of Flavocoxid (Limbrel) Versus Naproxen in Subjects With Moderate-Severe Osteoarthritis of the Knee” has shown the compound to have side effects comparable with placebo. The primary and secondary outcome measures in this trial included efficacy, safety, quality of life, and economic impact compared with the NSAID naproxen and placebo.

A related article in this issue of Current Rheumatology Reports will address the clinical trials that have been published to date, emphasising the major drawbacks of these studies. The general consensus is that most clinical trials conducted to date have been inadequately powered and have not been of sufficient length and duration.

## PubMed Literature Survey

A PubMed literature survey was conduced on 4 July 2012 using the keywords “herbal medicine” and “osteoarthritis”. This search revealed a total of 88 papers in the PubMed. Selected papers from this literature search are reviewed in this section.

In a double-blind study Mills et al. [60] studied the effects of a proprietary herbal medicine on the relief of chronic arthritis pain. Eighty-two subjects with chronic arthritis pain were randomly assigned for two months without crossover to either a product containing sarsaparilla, white willow bark, black cohosh, guaiacum resin, and poplar bark, a licensed over-the-counter (OTC) herbal medicine, or a placebo. Questionnaires revealed a mild analgesic effect in subjects with chronic arthritis.

Ginger, which originates from the dried or fresh root of the ginger plant, contains active ingredients that may have analgesic and anti-inflammatory properties. Bliddal et al. [61] conducted a randomized, placebo-controlled, crossover study of ginger extract and ibuprofen in patients with hip or knee OA. The effects of ginger extract were compared with placebo and ibuprofen in a controlled, double blind, double-dummy, cross-over study with a wash-out period of one week followed by three treatment periods in a randomized sequence, each of three weeks duration. As might be expected, the results of the study revealed the following ranking of efficacy: ibuprofen > ginger extract > placebo. No significant difference between placebo and ginger extract could be demonstrated in the crossover study. Statistically significant effects of ginger extract could only be demonstrated by complex statistical methods in the first period of treatment before crossover. No significant difference was observed in the study as a whole.

The efficacy of *Harpagophytum procumbens* in the treatment of knee and hip OA was investigated in a double-blind, randomized, multicentre clinical study [62]. The herbal medicine product was used at a dose of six capsules/day, each containing 435 mg of cryoground and powdered *Harpagophytum procumbens.* This product was compared with diacerhein 100 mg/day. Pain and functional disability were assessed by use of a visual analogue scale (VAS) and the severity of OA was evaluated by use of the Lequesne index. Although there was no difference in the efficacy of the two treatments, at the end of the study, patients taking the herbal product were using significantly less NSAIDs. The authors concluded that the herbal product was comparable with diacerhein in terms of efficacy but superior to diacerhein in terms of safety.

Choi et al. tested the effects of the herbal agent SKI 306X on proteoglycan degradation in rabbit cartilage explants in-vitro and a collagenase-induced rabbit model of OA [63]. SKI 306X is an extract of three herbs: *Clematis mandshurica*, *Trichosanthes kirilowii*, and *Prunella vulgaris*. The authors compared the effects of this herbal extract with those of dexamethasone and two NSAIDs, diclofenac and rofecoxib. Recombinant human interleukin-1α was used to induce proteoglycan (PG) degradation and the degree of PG degradation was assessed by measuring the quantity of glycosaminoglycans (GAGs) released into the culture medium. In-vivo experiments involved intra-articular injection of collagenase into the right knee joint of rabbits to induce OA-like changes followed by a histological examination after 28 days. SKI 306X inhibited PG degradation in a concentration-dependent manner. Interestingly dexamethasone, diclofenac, and rofecoxib did not suppress PG degradation. In-vivo studies showed that a 200-mg/kg dose of SKI 306X reduced OA-like histological changes, whereas diclofenac had no effect at 10 mg/kg. The authors concluded that the herbs in the SKI 306X extract have chondroprotective effects in-vitro and in-vivo.

Kim et al. [64] extended the work on SKI 306X by investigating its gastro-sparing effects on the gastric mucosa. They compared its effects with those of diclofenac, a conventional NSAID, and celecoxib, a cyclooxygenase-2 (COX-2)-specific inhibitor. To investigate the acute gastric damaging properties of SKI306X, the stomachs of the animals were histologically and immunohistochemically examined. Their results suggest that SKI 306X suppresses gastric leukotriene B_4_ (LTB_4_) synthesis without causing mucosal injury or diclofenac-induced gastric lesions. SKI 306X did not have a significant effect on the levels of prostaglandin E_2_ (PGE_2_). In addition, gastro-protective effects of SKI306X were induced by suppressing diclofenac-induced erosion and ulceration of gastric mucosa in a rat model, also by sparing the gastric mucosa by suppression of gastric leukotriene synthesis.

Teekachunhatean et al. [65] compared the Chinese herbal recipe Duhuo Jisheng Wan (DJW), a combination product containing *Angelica pubescens* and other plant extracts, with diclofenac for the symptomatic treatment of knee OA. This study was a randomized, double-blind controlled trial that included 200 subjects suffering from knee OA. Patients were evaluated after a run-in period of one week followed by weekly evaluations during subsequent weeks of treatment. Clinical assessments included VAS scores for pain and stiffness, Lequesne’s functional index, time for climbing up 10 steps, and physicians and patients’ overall opinions on improvement. In the first few weeks of treatment, the mean changes in VAS for walking pain, standing pain, and stiffness, and Lequesne’s functional index of the DJW group were significantly lower than those of the diclofenac group. However, the physicians’ and patients’ overall opinions did not significantly differ between the two groups and one third of patients in both groups experienced mild adverse events. The authors concluded that although DJW had clinical efficacy comparable with that of diclofenac, the slow onset of action and the adverse effects limited its clinical value as a treatment for knee OA.

Elegant studies by Wu et al. [66, 67•] have evaluated the anti-inflammatory activity of an ethanolic extract of *Caesalpinia sappan* in human chondrocytes and macrophages. *Caesalpinia sappan* is a common remedy in Traditional Chinese Medicine and possesses diverse biological activities. In the first study, the authors demonstrated the anti-inflammatory activity of *Caesalpinia sappan* extracts in an in-vitro model of joint inflammation. *Caesalpinia sappan* extracts inhibited the IL-1β-induced over-expression of inflammatory mediators at the transcriptional level in human chondrocytes and macrophages [67•]. In a subsequent study published a year later, the authors provided further molecular support for this observation [66]. They treated primary human chondrocytes (isolated from OA cartilage), SW1353 chondrosarcoma cells, and THP-1 macrophages with IL-1β or lipopolysaccharide (LPS). Nitric oxide (NO) and tumour necrosis factor-alpha (TNF-α) were evaluated by use of the Griess assay and ELISA, respectively. *Caesalpinia sappan* extracts dose-dependently inhibited expression of pro-inflammatory cytokines IL-1β and TNF-α in IL-1β-stimulated chondrocytes and LPS-stimulated THP-1 macrophages. *Caesalpinia sappan* extracts also suppressed the synthesis of NO in primary OA chondrocytes by blocking iNOS mRNA expression. They observed that the levels of IL1β-induced MMP-1, MMP-3, MMP-7, and MMP-9 mRNA, but not TIMP mRNA levels, were down-regulated in chondrocytes in response to *Caesalpinia sappan* extracts. Zymography suggested that *Caesalpinia sappan* extracts did not interfere with the proteolytic activity of MMP-2. Interestingly, the inhibition of COX-2 transcription by *Caesalpinia sappan* extracts was related to the inhibition of the p65/p50-driven transactivation of the COX-2 promoter. The authors concluded that *Caesalpinia sappan* extracts abrogate the IL-1β-induced over-expression of inflammatory mediators in human chondrocytes and macrophages. The most likely mechanism accounting for the anti-inflammatory activity of *Caesalpinia sappan* extracts is inhibition of NF-κB (p65/p50) signalling. The authors proposed that blocking IL-1β-induced NF-κB signalling and its downstream pro-inflammatory targets by *Caesalpinia sappan* extracts may reduce cartilage breakdown in arthritis.

Park and co-workers [68] examined the therapeutic effects of PG201, an ethanolic herbal extract in a collagenase-induced model of arthritis in rabbits. The right knees of rabbits were injected intra-articularly with collagenase and the rabbits were orally treated with distilled water, PG201 (200 mg/kg), or diclofenac (10 mg/kg) once a day for 8 weeks. Administration of PG201 significantly suppressed stiffness and joint space narrowing. Cartilage destruction and GAG release were substantially reduced in the knee joints. Expression of matrix metalloproteinases MMP-1, MMP-3, and MMP-13 was reduced by PG201. The levels of the inflammatory mediators IL-1β, PGE_2_, and nitric oxide NO were also reduced by PG201. The authors concluded that PG201 has therapeutic effects in this animal model of arthritis.

A study by Wang et al. [69] assessed the short-term efficacy and safety of Traditional Chinese herbal patches, Fufang Nanxing Zhitong Gao (containing, among other ingredients, extracts of *Rhizoma arisaematis, Radix aconite*, and *Flos caryophylli*) and Shangshi Jietong Gao, containing a 17-herb mixture, for painful knee OA. Patients were randomly enrolled in a double-blind, placebo-controlled study to receive the herbal patches, or a placebo patch for seven days. Outcome measures included VAS, Western Ontario, and McMaster Universities Osteoarthritis Index (WOMAC), and the Traditional Chinese Medicine Syndrome Questionnaire (TCMSQ) subscale. The study found no significant differences among the three groups in terms of short-term pain management.

Majima et al. [70] studied the effects of Boiogito, a Japanese herbal medicine containing an anti-inflammatory compound called sinomenin, on knee OA and joint effusion. Patients were randomly assigned to two groups, one receiving loxoprofen and Boiogito and the other group using loxoprofen only. Outcomes were evaluated over 12 weeks using knee scores and questionnaires. Knee scores was significantly improved in the group receiving Boiogito and loxoprofen compared with the loxoprofen only group. The authors concluded that Boiogito may be a useful treatment for knee OA.

Finally, a recent study from our laboratory attempted to characterize the anti-inflammatory mode of action of herbal extracts from rosehip (*Rosa canina*), willow bark (*Salix alba*), and nettle leaf (*Urtica dioica*) in an in-vitro model of primary canine articular chondrocytes [71]. The extract of willow bark has been used in traditional medicine as a pain reliever. The biological effects of the herbal extracts were studied in canine chondrocytes treated with IL-1β. Expression of collagen type II, proteoglycans, β1-integrin, SOX-9, COX-2, MMP-9, and MMP-13 was examined by western blotting. The extracts suppressed IL-1β-induced NF-κB activation by inhibition of IκBα phosphorylation, IκBα degradation, p65 phosphorylation, and p65 nuclear translocation. These events correlated with downregulation of NF-κB targets including COX-2 and MMPs. The extracts also reversed the IL-1β-induced downregulation of collagen type II, cartilage-specific proteoglycans (CSPGs), β1-integrin, and cartilage-specific transcription factor SOX-9 protein expression. We also used high-density cultures to demonstrate that the herbal extracts can stimulate new cartilage formation, even in the presence of IL-1β. The study concluded that herbal extracts can exert potent anti-inflammatory actions in-vitro and may have beneficial proanabolic effects on chondrocytes also. The observed reduction of IL-1β-induced NF-κB activation provides more evidence for natural targeting of NF-κB in arthritic diseases. In contrast to our work, a 2004 study concluded that extracts of willow bark have no relevant efficacy in patients with OA and RA [72].

## Regulatory Considerations

Herbal and alternatives medicines and nutraceuticals are bold challenges to government and state regulations. In 1992, the National Institutes of Health (NIH) established an Office of Alternative Medicine. The United States Congress subsequently elevated this to a Center for Complementary and Alternative Medicine with a multi-million dollar annual budget. Historically, the FDA [18] took the view that a product that made health claims was a drug. Consequently, in the 1970s vitamins and minerals were exempted from regulation as drugs as long as they did not make health claims. In the early 1980s, studies demonstrated that some food ingredients, for example fibre, provided specific benefits to health. Food manufacturers wanted to proclaim these benefits to consumers without having to obtain drug approval. The FDA wrestled with this problem until the US Congress passed the Nutrition Labeling and Education Act of 1990. This law authorized the FDA to issue regulations permitting specific health claims for foods, which led the agency to allow claims associating low levels of calcium with osteoporosis, dietary fats with cancer, and cholesterol with heart disease. After passage of the FDA Modernization Act of 1997, a food could make a health claim without FDA regulatory authority as long as the claim was based on an authoritative statement by a governmental or quasi- governmental scientific body (for example the NIH or the National Academy of Science [NAS]) and the agency was given advanced notice of the manufacturer’s intent. However, this leeway did not apply to dietary supplements and so it has resulted in much confusion. The presence of metals, for example lead, mercury, and arsenic in some herbal remedies makes them potentially very harmful. Furthermore, some of the ingredients can interact with each other and with conventional medications that patients may be taking.

The situation in Europe has been equally chaotic. Consequently the European Food Safety Authority (EFSA) based in Parma, Italy, has issued new guidelines and proposed new scientific requirements for health claims related to the maintenance of joints and to the reduction of the risk of developing OA. The EFSA has proposed that clinical trials of functional foods and nutraceuticals should be designed in new and innovative ways to demonstrate a “beneficial physiological effect” on healthy joints. According to these new guidelines, only clinical trials designed to demonstrate a beneficial physiological effect on joints or a reduction in joint degradation in people without OA should be accepted as indicative. These guidelines present some major new challenges to the scientific and clinical communities. Furthermore, they create a number of opportunities for new types of clinical trial. Studies performed in non-diseased (but including high risk) population subgroups in which the incidence of OA is the outcome measure could be used for substantiation of health claims relating to the normal maintenance of the joint. Whilst attempting to address these requirements, we need to discriminate between food and non-food supplements. Studies dealing with “non-foods” will require a much more traditional pharmacological design compared with studies on “foods”. Clearly, addressing these issues requires new strategies and large-scale clinical studies lasting several decades. Such new trials will require radical rethinking of the concept of clinical trials in the OA research community. Human studies seem to be central to substantiation of clinical data and study groups should be representative of the entire population. Hierarchy of evidence is also considered; for example, interventional studies are of greater significance than observational studies and reproducibility of the effect much be demonstrated. In addition, demonstrating efficacy of food supplements to the EFSA will also require data on tolerance and safety, specifically gastric tolerance, hepatotoxicity, renal toxicity, and allergenicity.

## Hormetic Effects of Herbal Medicines

Herbal medicines may have beneficial effects at low concentrations as long as they are consumed over a long period of time. Studying this aspect of their action may require longer-term studies. The term “hormesis” is used to describe a biphasic dose response to an environmental agent or chemical characterized by stimulation or beneficial effects at low doses or toxic and inhibitory effects at high doses. Hormesis is a fundamental concept that applies to the almost all drugs derived from plants and microorganisms. Even synthetic drugs thought to act on a specific molecular target may exert “off-target” or “hormetic” effects. The response of the cell or organism to the low dose of a toxin is regarded as an adaptive compensatory process after initial disruption in homeostasis. Thus, a short working definition of hormesis is “a process in which exposure to a low dose of a chemical agent or environmental factor that is damaging at higher doses induces an adaptive beneficial effect on the cell or organism”. The prevalence in the literature of hormetic dose responses to environmental toxins has been reviewed comprehensively [73], as have the implications of toxin-mediated hormesis for understanding carcinogenesis and its prevention [74]. Several different terms are commonly used to describe specific types of hormetic responses including “preconditioning” and “adaptive stress response”. Hormesis in aging is defined as the life-supporting beneficial effects resulting from the cellular responses to single or multiple rounds of mild stress. Thus, hormesis may also have the capacity to modify the ageing process [75]. The dose–response relationships for herbal medicines commonly have the same hormetic dose–response relationships as their toxic counterparts. Many agents, for example antibacterials, antifungals, antivirals, and tumour-fighting drugs, have hormetic dose responses. Even curcumin exerts a hormetic effects at the cellular and molecular levels in mammalian cells [76]. Therefore, we need to consider the potential long-term hormetic effects of herbal medicines.

## Conclusions

**Fig. 2:**
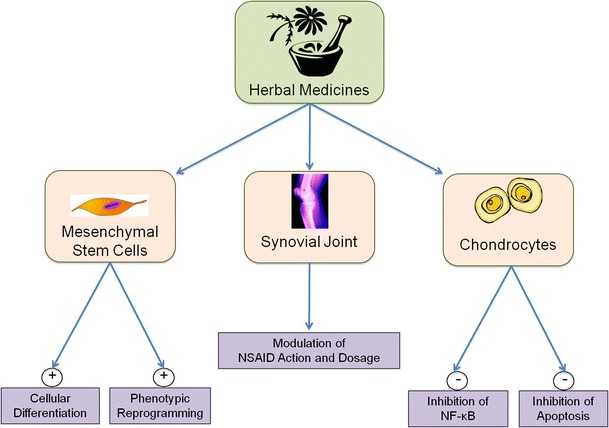
Schematic diagram summarizing some of the effects of herbal medicines on chondrocytes, synovial tissues, and mesenchymal stem cells (MSCs)

Herbal remedies, and dietary supplements have become important areas of research and clinical practice in orthopaedics and rheumatology [77]. Therefore, it is important that health-care providers and patients are aware of the evidence for or against these approaches [78]. Some of the published evidence suggests that several herbal medicines and dietary supplements have the capacity to alleviate the pain of OA and RA [79]. For several treatments, the risk–benefit profile is encouraging. Some herbal remedies are inhibitors of NF-κB and may be able to reduce the consumption of NSAIDs and stimulate the differentiation of MSCs (Fig. 2). The author’s personal view is that only some of the research on herbal medicines can be defined as “rigorous”. A substantial proportion of the published literature does not reach the required levels of scientific rigor. For example, some studies on herbal and traditional medicines have not included a placebo group [80]. The first preliminary study on the anti-rheumatic activity of curcumin suffered from fundamental weaknesses relating to experimental design [81]. Many other studies suffer from methodological limitations [82]. Clearly, more rigorous testing of herbal remedies and complementary treatments should be the main priority of future studies.

The main purpose of herbal and complementary medicines is to supplement some of the benefits from existing pharmaceutical treatment modalities [83]. The objective is to reduce the frequency of consumption and dosages of conventional drugs, for example NSAIDs. The objective is not to replace NSAIDs altogether because they not only provide pain relief, but also have valuable anti-inflammatory activity. However, elderly patients with OA routinely use prescribed and alternative products at the same time. There is potential for adverse drug interactions and patients should be made aware of the risks associated with taking multiple products [84].

In summary, the elderly population is rapidly growing and expanding throughout the developed and developing worlds. Therefore, the use of herbal and complementary medicines for treatment of persistent musculoskeletal pain will continue to increase [85]. When we consider the popularity of herbal remedies, more mechanistic basic studies should be encouraged as a prelude to large-scale, randomized clinical trials. In addition, basic common sense must prevail throughout this process. For instance, is it realistic to propose that an herbal extract tested in-vitro for inhibition of COX-2 can actually be effective when ingested by human beings? Is it not prudent to consider gastrointestinal absorption, systemic processing, and bioavailability of the active ingredient(s)? This is the only way to address the mismatch between what we had hoped for with herbal medicines (efficacy with more safety and less toxicity) and what we actually have so far. This is why more rigorous clinical trials examining the efficacy of herbal remedies are needed before definitive recommendations regarding the application of these modalities can be made [85]. Our research efforts must be directed toward defining the risk-to-benefit ratio for herbal and alternative medicines for specific musculoskeletal conditions [82].
